# Supplementary material for: In Vitro Monitoring of Babesia microti Infection Dynamics in Whole Blood Microenvironments
Source: Adv Sci (Weinh). 2025 Aug 11;12(41):e08185. doi: 10.1002/advs.202508185 (PMC12591138; doi:10.1002/advs.202508185)
Supplement: Supplementary file 1 — Supporting Information [file ADVS-12-e08185-s001.docx]

Supporting Information

***In Vitro* Monitoring of *Babesia microti* Infection Dynamics in Whole Blood Microenvironments**

*Chao Li, Emily G. Bache, Amy L. Apgar, Danielle M. Tufts^†^*, and Tagbo H.R. Niepa^†^**

**This SI file includes:**

Figures S1 to S6

Table S1

Movies S1 and S2


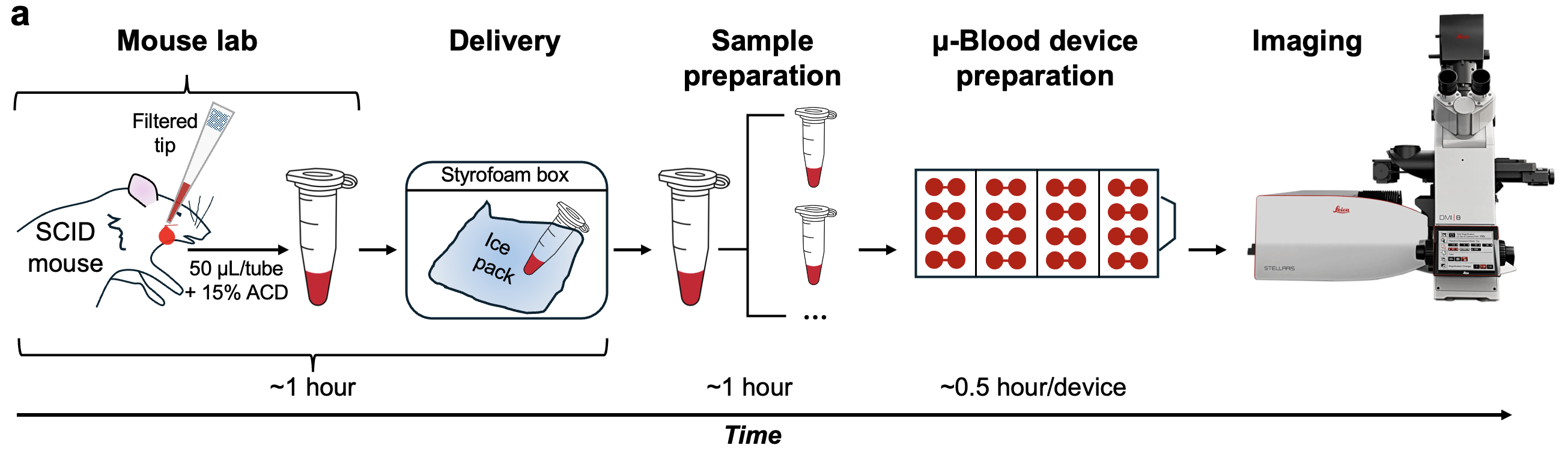


**
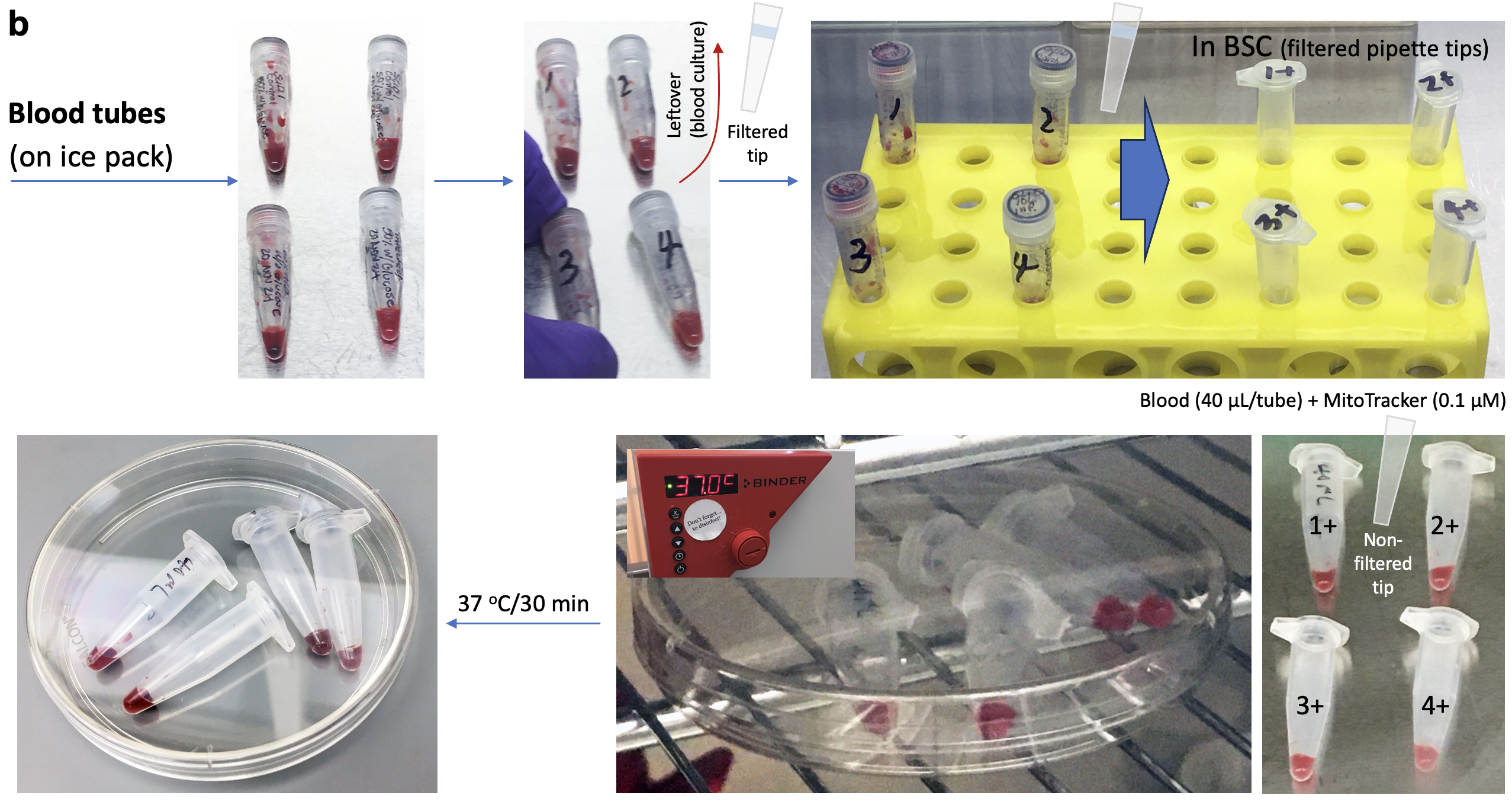
**

**
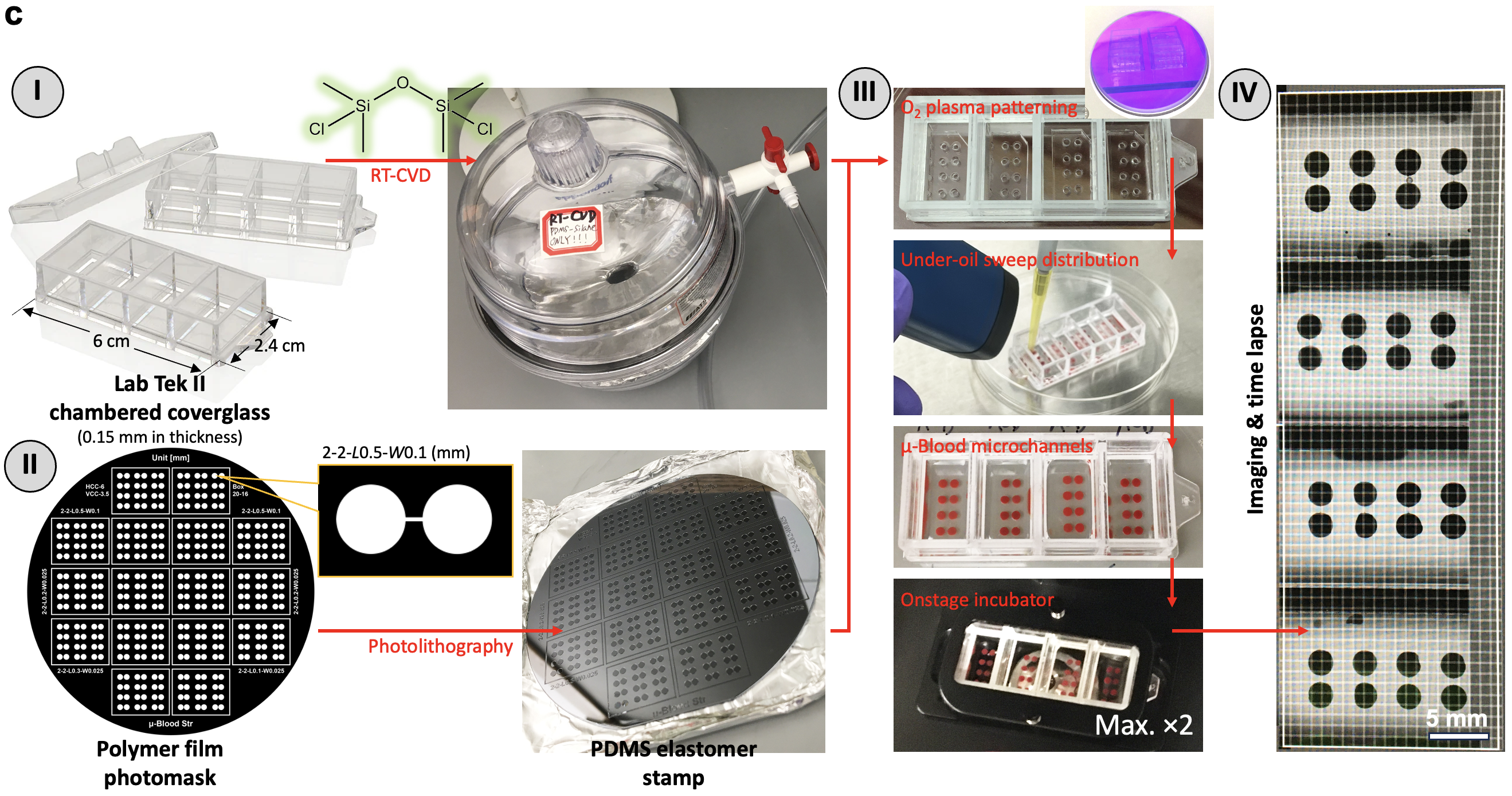
**

**Figure S1.** Workflow of μ-Blood *Babesia* assay. a) Average time use in each step from blood collection to data collection. b) Representative blood sample preparation before sample loading on the device. μ-Blood can handle small blood sample volume for 10+ μL. In each experiment, the leftover blood was put in culture with LB media as bacterial contamination control. c) μ-Blood device fabrication and operation including I – PDMS silane CVD (done independently before the assay), II – PDMS stamp preparation (done independently before the assay, stamps reusable), III – surface patterning and sample loading (maximum of two μ-Blood devices at a time on microscope), and IV – imaging and time lapse (a representative preview of the entire device, rotated 90^o^ counterclockwise). μ-Blood device preparation in (a) corresponds to III in (c). Imaging in (a) corresponds to IV in (c).


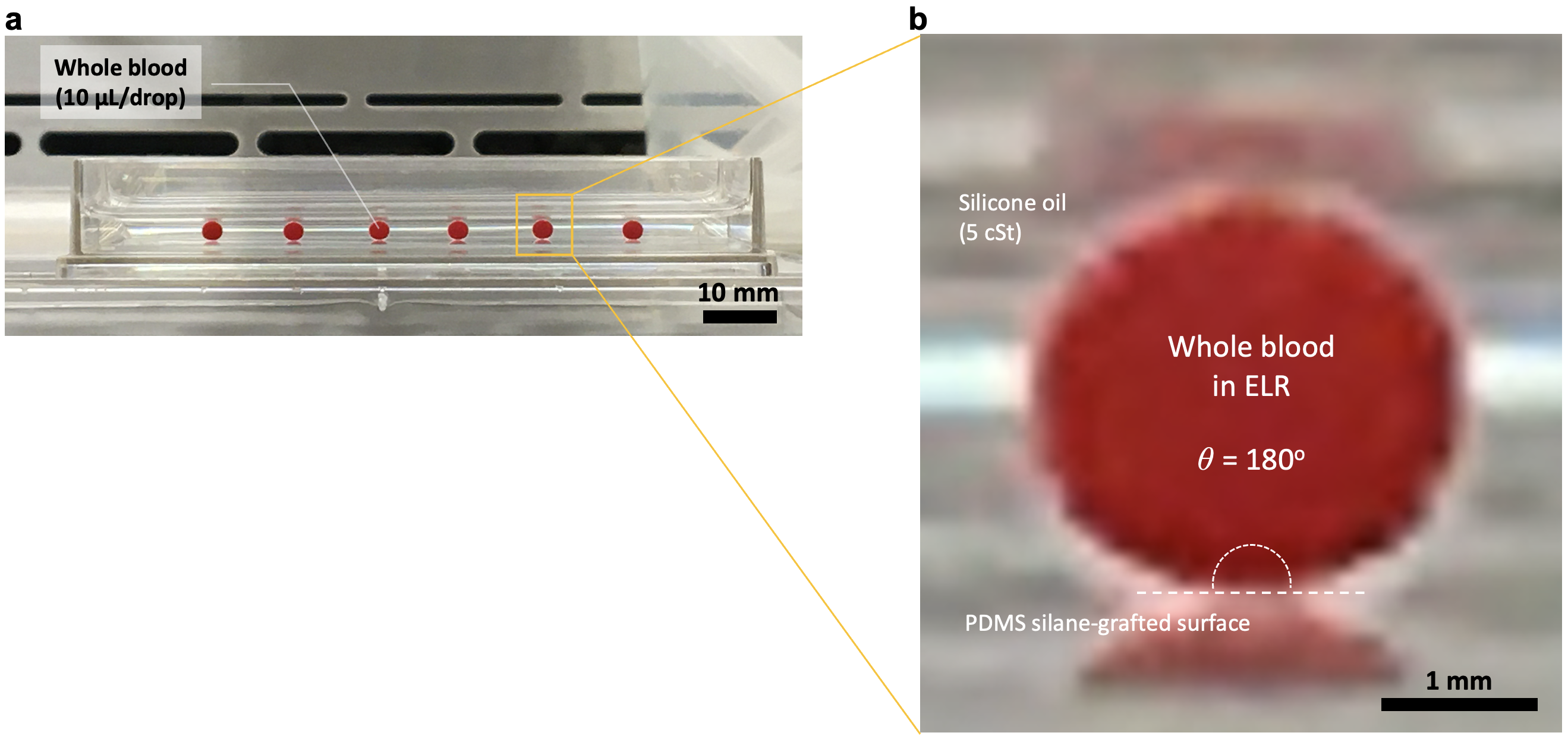


**Figure S2.** ELR of whole blood on PDMS silane-grafted glass surface under oil (silicone oil, 5 cSt). a) Six drops of whole blood (sheep, defibrinated, 10 μL per drop) in a Nunc OmniTray. b) Zoomed-in picture of ELR blood drop shows the droplet profile with a Young’s contact angle *θ* = 180^o^.


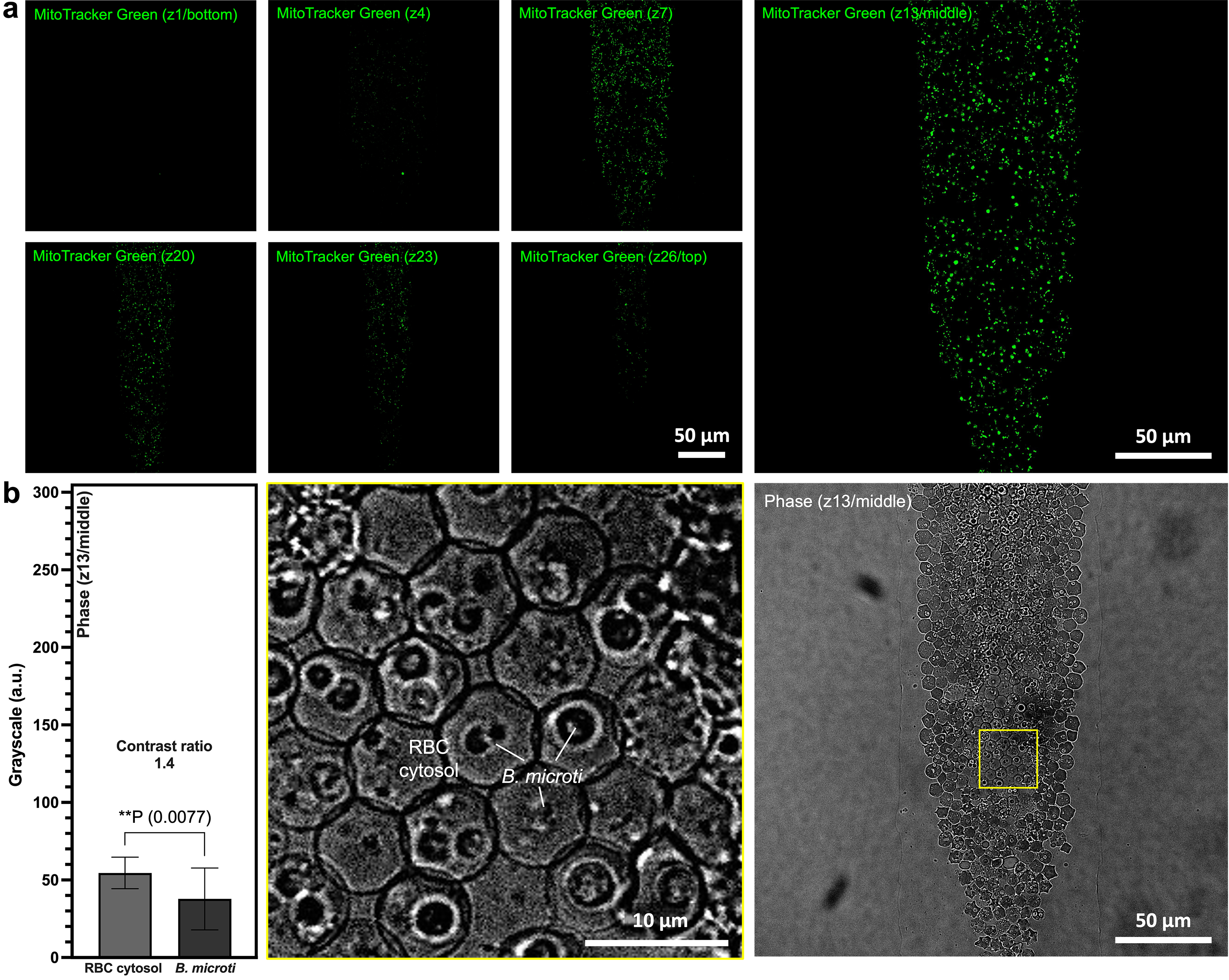


**
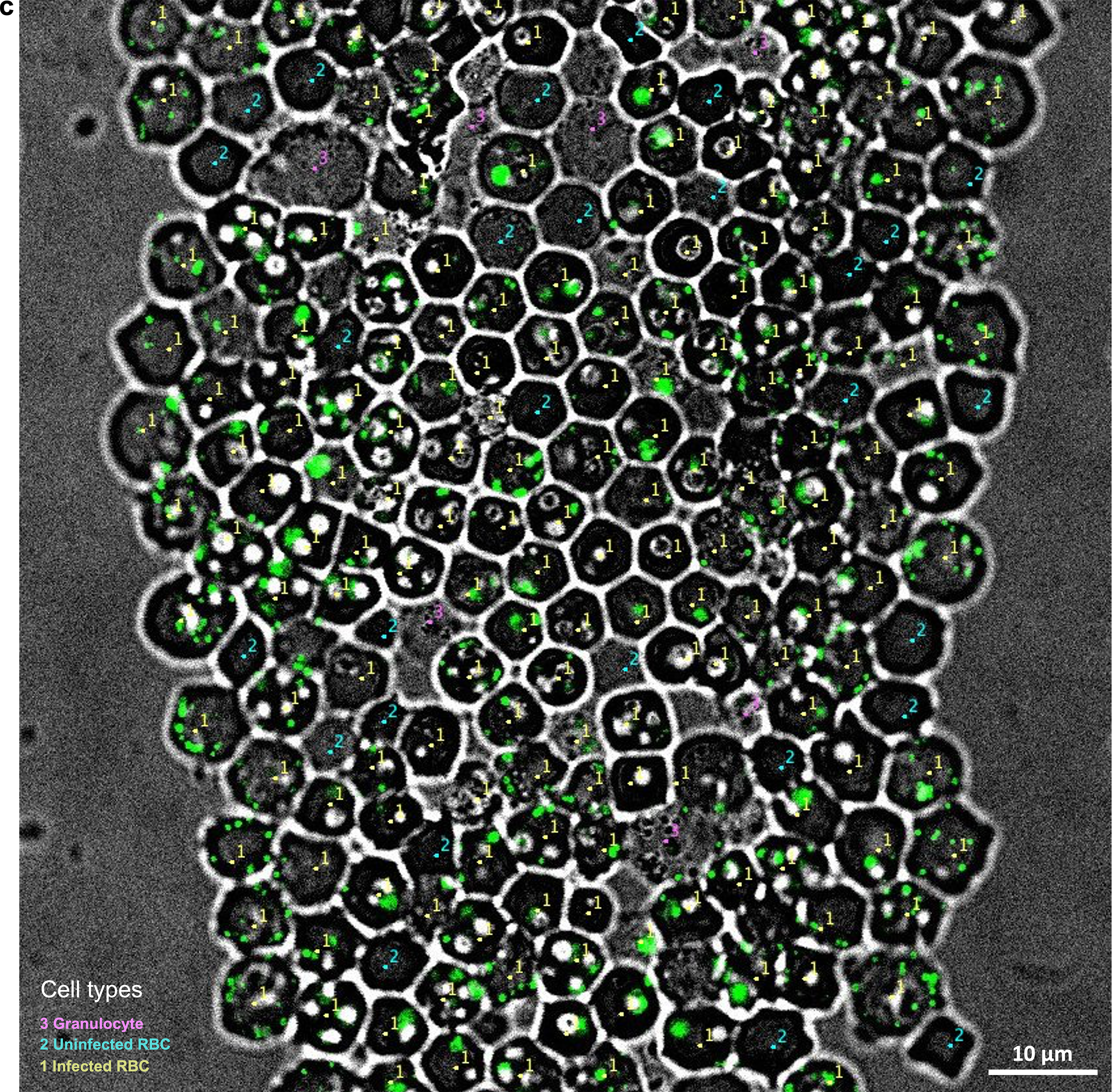
**

**Figure S3.** Confocal images and z-stack slices of *B. microti*-infected SCID mouse blood on μ-Blood. a) Selected MitoTracker Green images from z-stack in Figure 2. The middle slice of MitoTracker Green shows the maximum fluorescence intensity and coverage of mitochondria. b) The middle slice of phase from z-stack shows the minimum contrast ratio (1.4) between RBC cytosol and *B. microti* compared to the bottom (44.6) and top (37.3) slice (Figure 2e). A paired two-tailed t-test was used to calculate *p*-values for these comparisons; error bars are mean ± s.d. ***p* ≤ 0.01. c) A representative image of *B. microti*-infected blood with the cell-count markers. Cell types include 1. Infected RBC, 2. Uninfected RBC, and 3. Granulocyte.


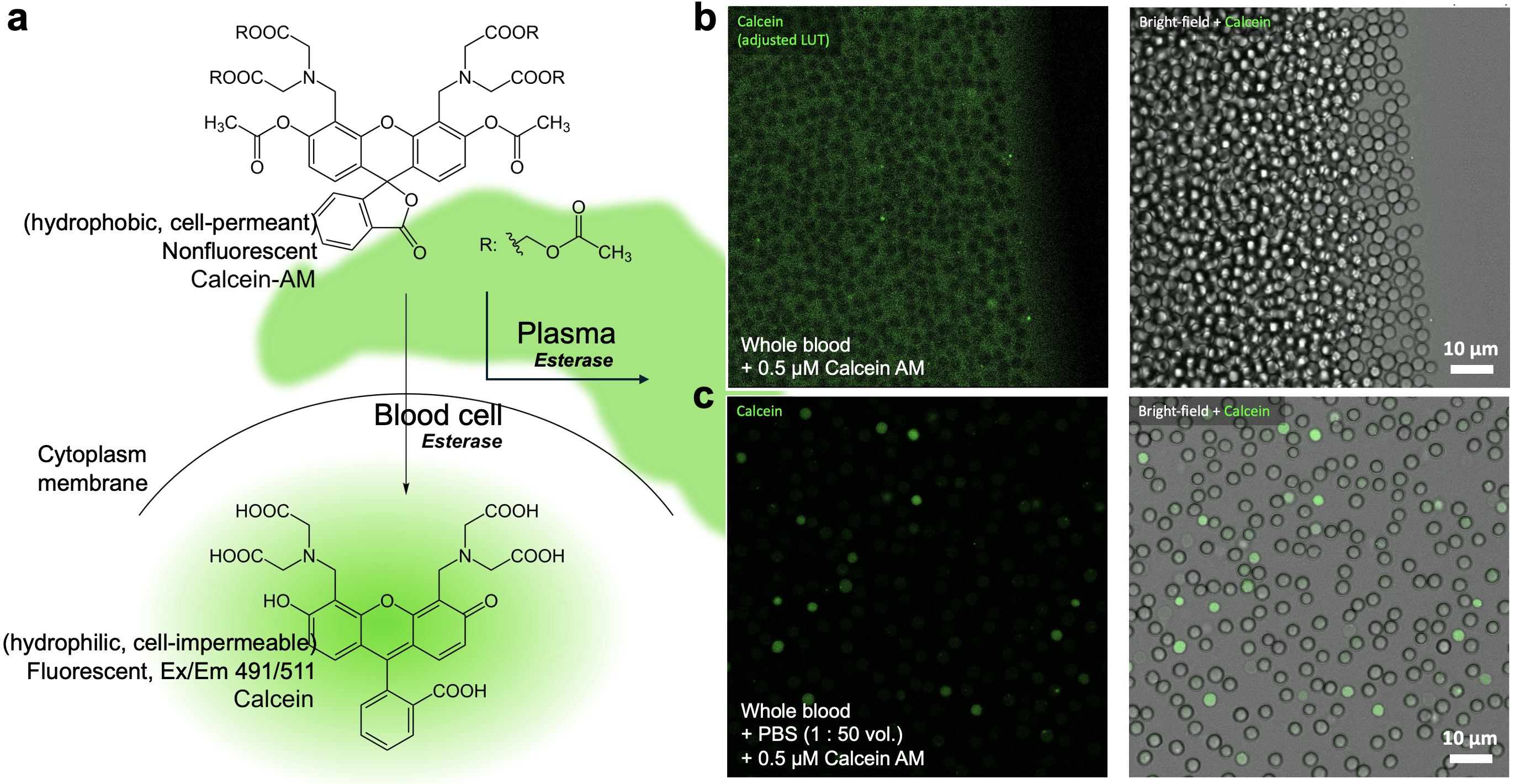


**Figure S4.** Calcein AM cell viability stain in *B.* microti-infected whole blood. a) Schematic shows the molecular mechanism of Calcein staining. b) High fluorescence background and the flipped contrast between plasma (green/bright) and blood cells (dark) due to the hydrolysis of Calcein AM in plasma. c) Low fluorescence background and the normal contrast between plasma (dark) and blood cells (green/bright) after PBS dilution of whole blood (1:50 vol.).


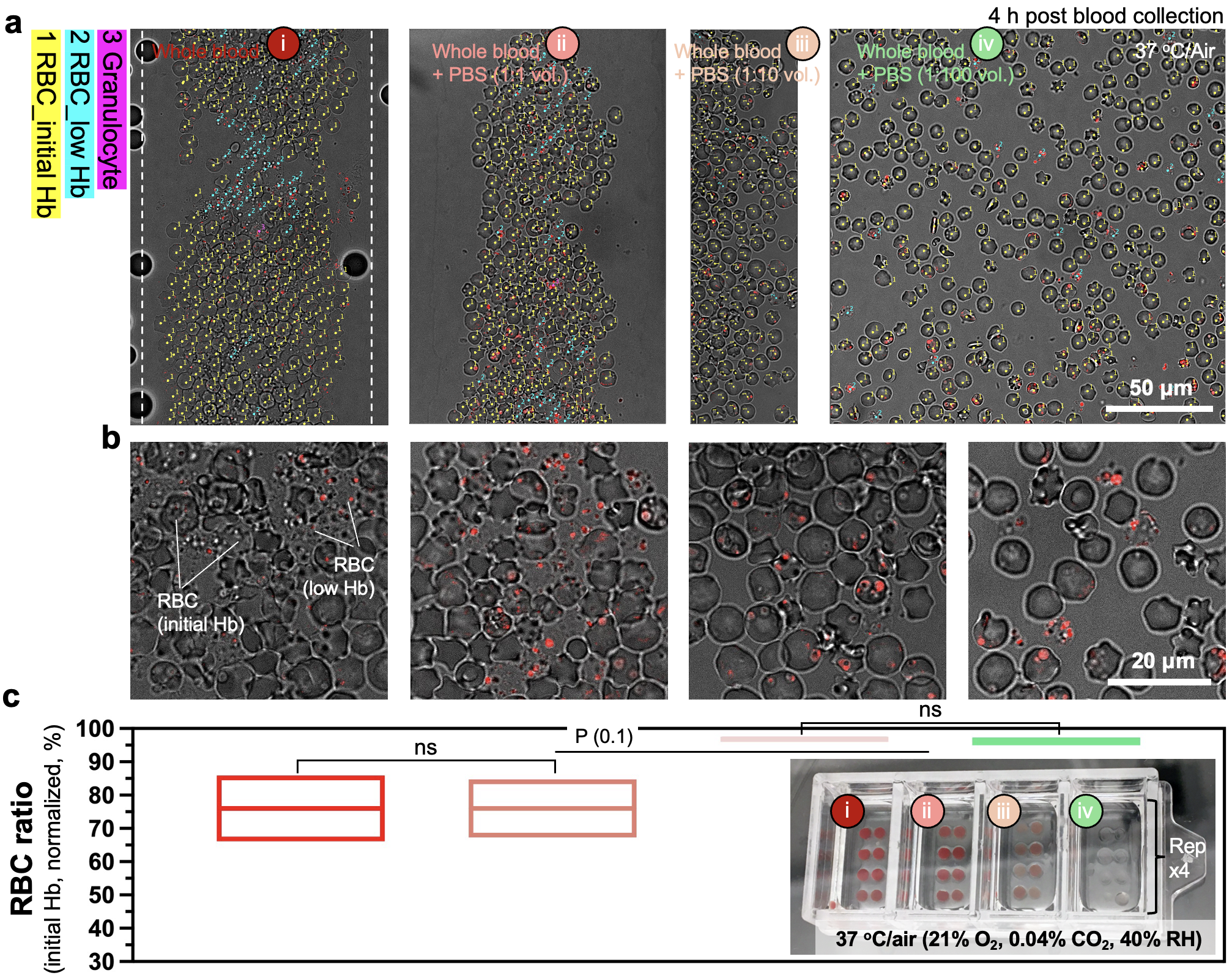


**
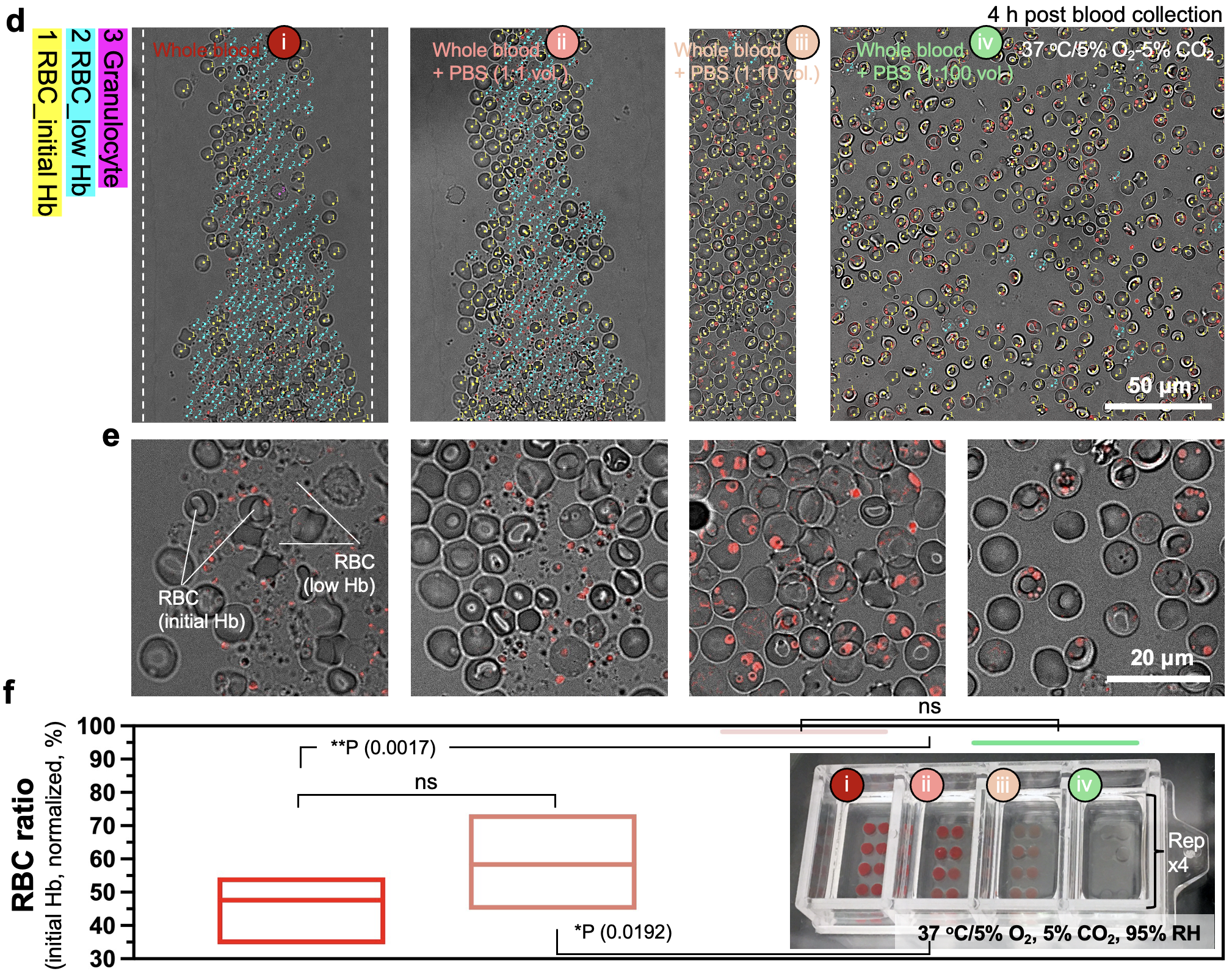
**

**Figure S5.** Hb reduction of *B. microti*-infected RBCs in different culture environments. a) Cell counts from four culture conditions including i – whole blood (without PBS dilution), ii – whole blood + PBS (1:1 vol. dilution), iii – whole blood + PBS (1:10 vol. dilution), and iv – whole blood + PBS (1:100 vol. dilution) in ambient air (37 ^o^C, air – 21% O_2_, 0.04% CO_2_, ambient <40% RH). The cell count was performed with three cell types including 1. RBC with initial Hb, 2. RBC with low Hb, and 3. Granulocyte. b) Zoomed-in images show the RBC Hb level (grayscale) and *B. microti* (MitoTracker Orange) corresponding to each condition in (a). c) Comparison of the ratio of RBCs with initial Hb through the four culture conditions at 4 h post blood collection. d), e), and f) The corresponding results of the four culture conditions with the samples cultured in a CO_2_ incubator (37 ^o^C, 5% O_2_, 5% CO_2_, 95% RH). The whole blood in this test was from the same blood sample collected from a *B. microti*-infected SCID mouse. Insets in (c) and (f) showed the μ-Blood devices in this test. A one-way ANOVA Tukey’s multiple comparison test was used to calculate *p*-values; error bars are mean ± s.d. **p* ≤ 0.05, ***p* ≤ 0.01, and ns – not significant.

**Figure S6.** Effect of blood dilution on Hb reduction of *B. microti*-infected RBCs. a) Composite images (phase + MitoTracker Orange) show the change of Hb level in *B. microti*-infected RBCs (yellow numbers) in whole blood (without dilution) through 4 days. b) Composite images (phase + MitoTracker Orange) show the change of Hb level in *B. microti*-infected RBCs in whole blood + PBS (1:10 vol. dilution) through 4 days.

**Table S1.** Parasitemia level measured with blood smear and μ-Blood.

| **Mouse ID** | **Date of blood collection** | **Parasitemia level (blood smear w Wright-Giemsa stain)^a)^** | **Parasitemia level (μ-Blood)** | **Corresponding figure** |
| --- | --- | --- | --- | --- |
| SCID106 | 2024-10-15 | 59.8 ± 3.8% | 82.2 ± 4.6% | Figure 2 |
| SCID106 | 2024-11-20 | 79.7 ± 5.7% |  | Figure 3 |
| SCID106 | 2024-12-18 | 83.0 ± 2.6% |  | Figure 5 |

^a)^ Blood smear comes with limited accuracy and consistency, especially on distinguishing infected RBCs with a single parasite cell from uninfected RBCs (Figure. 2f).

**Movie S1.** Phase contrast flip from confocal z-stack imaging. 5.2 s per frame_ FPS6_31.2× speed_2.25 min z-stack from bottom (z1) to top (z26).

**Movie S2.** Hb reduction of a *B. microti*-infected RBC. 5 min per frame_FPS12_3600× speed_10 h time lapse.
